# PRMT5 critically mediates TMAO-induced inflammatory response in vascular smooth muscle cells

**DOI:** 10.1038/s41419-022-04719-7

**Published:** 2022-04-04

**Authors:** He Liu, Kunpeng Jia, Zhengnan Ren, Jia Sun, Li-Long Pan

**Affiliations:** 1 School of Medicine and School of Food Science and Technology, Jiangnan University, 214122 Wuxi, P. R. China; 2 State Key Laboratory of Food Science and Technology, Jiangnan University, 214122 Wuxi, P. R. China

**Keywords:** Methylation, Vascular diseases

## Abstract

A high plasma level of the choline-derived metabolite trimethylamine N-oxide (TMAO) is closely related to the development of cardiovascular disease. However, the underlying mechanism remains unclear. In the present study, we demonstrated that a positive correlation of protein arginine methyltransferase 5 (PRMT5) expression and TMAO-induced vascular inflammation, with upregulated vascular cell adhesion molecule-1 (VCAM-1) expression in primary rat and human vascular smooth muscle cells (VSMC) in vitro. Knockdown of PRMT5 suppressed VCAM-1 expression and the adhesion of primary bone marrow-derived macrophages to TMAO-stimulated VSMC. VSMC-specific PRMT5 knockout inhibited vascular inflammation with decreased expression of VCAM-1 in mice. We further identified that PRMT5 promoted VCAM-1 expression via symmetrical demethylation of Nuclear factor-κB p65 on arginine 30 (R30). Finally, we found that TMAO markedly induced the expression of nicotinamide adenine dinucleotide phosphate oxidase 4 (Nox4) and production of reactive oxygen species, which contributed to PRMT5 expression and subsequent VCAM-1 expression. Collectively, our data provide novel evidence to establish a Nox4-PRMT5-VCAM-1 in mediating TMAO-induced VSMC inflammation. PRMT5 may be a potential target for the treatment of TMAO-induced vascular diseases.

## Introduction

Cardiovascular diseases, the major cause of morbidity and mortality worldwide, consist of a group of disorders of the heart and blood vessels [1]. Accumulating evidence suggests that the inflammation of vascular smooth muscle cells (VSMC) is a basic pathological feature of vascular dysfunction associated with vascular diseases [2, 3]. Recent insights into the pathogenesis of inflammatory vascular diseases underscore the importance of induced expression of proinflammatory adhesion molecules, such as vascular cell adhesion molecule-1 (VCAM-1) and intracellular adhesion molecule-1, which occurs in VSMC [2]. Plasma trimethylamine N-oxide (TMAO), a metabolite of the dietary lipid phosphatidylcholine, has been proven to be associated with an increased risk of major cardiovascular diseases in both human and animal-model studies [4, 5]. TMAO has been demonstrated to activate protein kinase-like ER kinase and enhance reactive oxygen species (ROS) generation in VSMC, which augmented angiotensin II -induced vasoconstriction [6]. However, a clear mechanistic link between TMAO and vascular inflammation is not yet established. Identifying the mechanisms underlying TMAO-induced VSMC inflammation is a crucial step toward the development of effective therapeutics.

Epigenetic modulators have been shown to play a major role in the regulation of vascular, immune, and tissue-specific gene expressions within atherosclerotic lesions [7]. Protein arginine methyltransferase 5 (PRMT5), the primary type II histone methyltransferase, contains a triosephosphate isomerase-barrel fold and possesses histone symmetric demethylase activity [8]. Previous studies have indicated that PRMT5 participates in numerous pathophysiological processes through modulating cell proliferation [9], inflammation [10], and tumor formation [11]. PRMT5 has also been implicated in epigenetic regulation of cell adhesion by transcriptional repression of the E-cadherin gene [12]. Despite recent advances on its role in endothelial inflammation [13], whether PRMT5 is involved in modulating VSMC inflammation remains elusive.

Here, we reported for the first time that aberrant PRMT5 overexpression positively correlated with TMAO-induced vascular inflammation. Mechanically, PRMT5 post-translationally methylated nuclear factor-κB (NF-κB) p65 subunit, a step imperative to the VCAM-1 induced by TMAO. Furthermore, we found that the TMAO-induced PRMT5 expression was mediated at least in part through upregulation of Nox4 and subsequent production of H_2_O_2_. Elucidating the role of TMAO and its underlying mechanisms during VSMC inflammation may provide new insights into the prevention and treatment of TMAO-related cardiovascular events.

## Results

### TMAO induces PRMT5 and VCAM-1 expression in VSMC

**Fig. 1:**
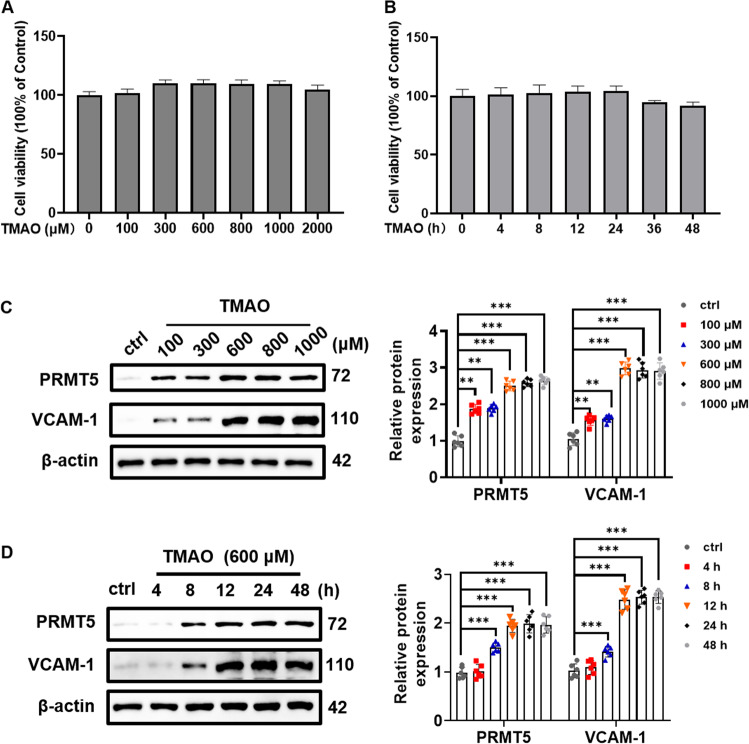
TMAO upregulates PRMT5 and VCAM-1 expression in rat VSMC. **A** VSMC were treated with different concentrations of TMAO (100–2000 μM) for 24 h. Subsequently, cell viability was determined using 3-(4, 5-dimethylthiazol-2-yl)-2, 5-diphenyl tetrazolium bromide (MTT) assay and quantitative analysis of cell viability. **B** VSMC were treated with TMAO (600 μM) for indicated periods (4–48 h), and cell viability was detected using MTT assay and quantitative analysis of cell viability. **C** VSMC were treated for indicated concentrations of TMAO for 24 h. Representative blots and quantitative analysis of VCAM-1 and PRMT5. The protein expression level was normalized to β-actin. **D** VSMC were treated with TMAO (600 μM) for indicated periods. Representative blots and quantitative analysis of VCAM-1 and PRMT5. The protein expression level was normalized to β-actin. Data shown are means ± SD, *n* = 6 per group from three independent experiments. **P < 0.01, ***P < 0.001 using a one-way ANOVA followed by Tukey’s post hoc test.

To evaluate potential cytotoxic effect of TMAO on primary rat VSMC, the cells were incubated with TMAO (100–2 000 μM) for 24 h or incubated with TMAO (600 μM) for the indicated periods and cell viability was evaluated by MTT assay. As shown in Fig. 1A, B, TMAO at the concentration range from 100 to 2000 μM did not affect the cell viability, indicating non-cytotoxic effect of TMAO observed under above condition. Importantly, TMAO (100–1000 μM) treatment dose-dependently induced PRMT5 and VCAM-1 expression in primary cultures of rat and human VSMC (Fig. 1C and Suppl. Fig. 1A). In addition, treatment of both primary rat and human VSMC with TMAO (600 μM) induced PRMT5 and VCAM-1 expression in a time dependent manner (Fig. 1D and Suppl. Fig. 1B). Taken together, these results indicate that upregulation of PRMT5 is correlated with TMAO-induced VCAM-1 expression in VSMC.

### PRMT5 positively regulates TMAO-induced VCAM-1 and inflammatory cytokine expression in VSMC

**Fig. 2:**
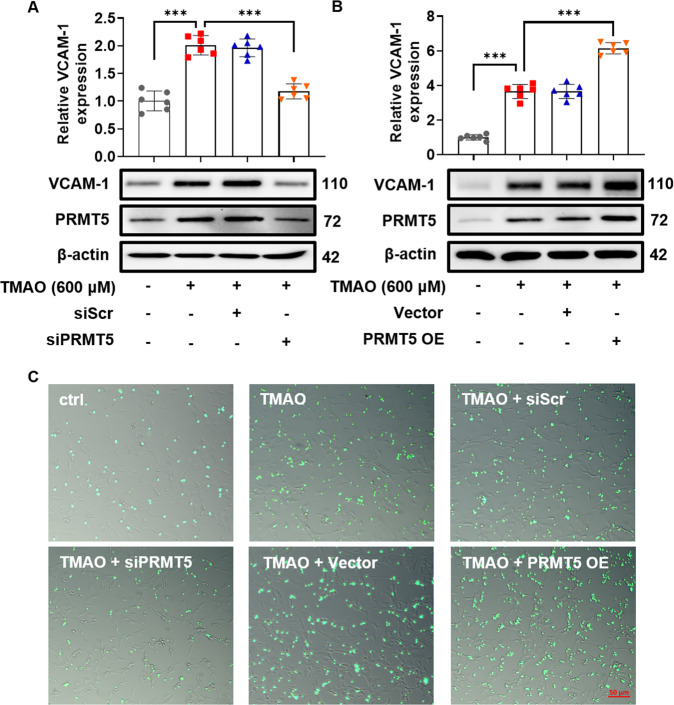
PRMT5 is responsible for TMAO-induced VCAM-1 expression. VSMC were transfected with PRMT5 siRNA (siPRMT5) or PRMT5 cDNA (PRMT5 OE), then stimulated with TMAO (600 μM) for 24 h. **A**, **B** Representative blots and quantitative analysis of VCAM-1 and PRMT5. The protein expression level was normalized to β-actin. **C** Representative images obtained by fluorescence microscope show cell adhesion. Data shown are means ± SD, *n* = 6 per group from three independent experiments. ***P < 0.001 using a one-way ANOVA followed by Tukey’s post hoc test.

Next, the role of PRMT5 on VCAM-1 expression in TMAO-stimulated VSMC was investigated. As shown in Fig. 2A and Suppl. Fig. 2, PRMT5 siRNA, but not scrambled siRNA, dramatically diminished TMAO-induced VCAM-1 expression in both primary rat and human VSMC. As expected, PRMT5 overexpression by transfection with a PRMT5 cDNA plasmid aggravated TMAO-induced VCAM-1 expression (Fig. 2B). Consistent with the results on the expression of VCAM-1, adhesion of BMDM was remarkably increased when VSMC were stimulated with TMAO, which was significantly attenuated by PRMT5 siRNA, but not scrambled siRNA. Conversely, PRMT5 overexpression by plasmid transfection further promoted the adhesion of BMDM to VSMC (Fig. 2C). Furthermore, transfection with PRMT5 siRNA, but not scrambled siRNA, dramatically reduced TMAO-induced mRNA expression of pro-inflammatory cytokines including IL-6 and IL-1β (Suppl. Fig. 3). Taken together, our results demonstrate that PRMT5 is critical for TMAO-induced VCAM-1 and pro-inflammatory cytokine expression in VSMC.

### PRMT5 promotes TMAO-induced VCAM-1 expression via methylation of NF-κB p65

**Fig. 3:**
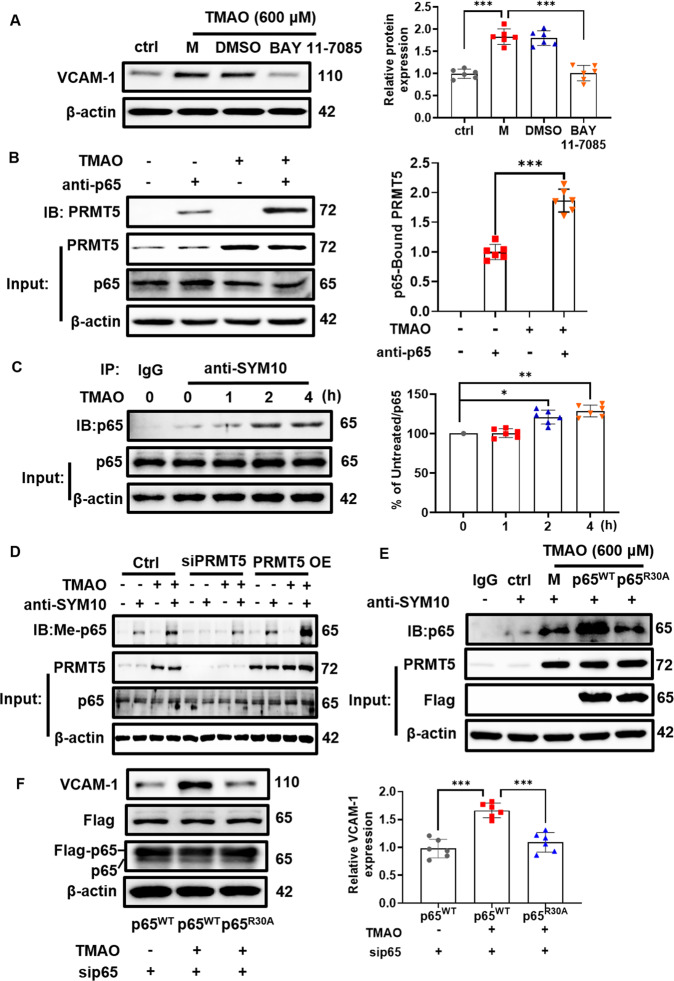
PRMT5-mediated NF-κB p65 methylation is involved in TMAO-stimulated VCAM-1 expression in VSMC. **A** VSMC were pretreated with BAY 11-7085 for 4 h and then stimulated with TMAO (600 μM) for 24 h. Representative blots and quantitative analysis of VCAM-1. The protein expression level was normalized to β-actin. **B** VSMC were treated with TMAO (600 μM) for indicated periods. Co-immunoprecipitation analysis of the interaction between PRMT5 and p65. **C** VSMC were treated with TMAO (600 μM) for 24 h. Representative blots of arginine methylation of p65. Immunoprecipitated SYM10 was blotted with an antibody against p65. **D** Representative blots of arginine methylation of p65. Immunoprecipitated SYM10 was blotted with an antibody against p65 in VSMC transfected with PRMT5 siRNA (siPRMT5) or PRMT5 cDNA plasmid (PRMT5 OE) followed by TMAO (600 μM) stimulation. **E** Flag-tagged p65 wide-type (p65^WT^) or R30A mutant (p65^R30A^) plasmid was transfected to VSMC, and then treated with TMAO (600 μM). Representative blots of arginine methylation of p65. Immunoprecipitated SYM10 was blotted with an antibody against p65. **F** Flag-tagged p65 wide-type (p65^WT^) or R30Amutant (p65^R30A^) plasmid was transfected to VSMC, and then treated with TMAO (600 μM). Representative blots and quantitative analysis of VCAM-1. The protein expression level was normalized to β-actin. Data shown are means ± SD, *n* = 6 per group from three independent experiments. *P < 0.05, **P < 0.01, ***P < 0.001 using a one-way ANOVA followed by Tukey’s post hoc test.

Activation of NF-κB is crucial for driving VCAM-1 gene expression [14]. To examine whether PRMT5 enhanced TMAO-induced VCAM-1 expression through modulating NF-κB p65 activation, VSMC were pretreated with a specific NF-κB inhibitor BAY 11-7085. As shown in Fig. 3A, BAY 11-7085 mitigated TMAO-stimulated VCAM-1 expression. Co-immunoprecipitation analysis further demonstrated that the interaction between PRMT5 and NF-κB p65 subunit was significantly increased in TMAO-stimulated VSMC (Fig. 3B). To further explore how PRMT5 affected the activation of NF-κB p65, the arginine methylation levels of NF-κB p65 were analyzed by co-immunoprecipitation with anti-symmetrical dimethylarginine (SYM-10) antibody. As shown in Fig. 3C, TMAO stimulation caused symmetrical dimethylation of NF-κB p65 in a time dependent manner. PRMT5 silencing markedly reduced the level of arginine dimethylation of NF-κB p65, while PRMT5 overexpression further enhanced the level of arginine dimethylation of NF-κB p65 in TMAO-stimulated VSMC (Fig. 3D). In addition, overexpressing NF-κB p65, but not NF-κB p65 R30A mutant, significantly increased the level of arginine dimethylation of NF-κB p65 in TMAO-stimulated VSMC (Fig. 3E). To evaluate the effect of NF-κB p65 R30A mutation on the expression of VCAM-1, endogenous NF-κB p65 was knocked down by siRNA and then transfected with wild-type cDNA or mutant p65 cDNA (R30A). As shown in Fig. 3F, overexpressing wild-type NF-κB p65 cDNA, but not NF-κB p65 R30A mutant, significantly increased the level of VCAM-1 expression in TMAO-stimulated VSMC.

**Fig. 4:**
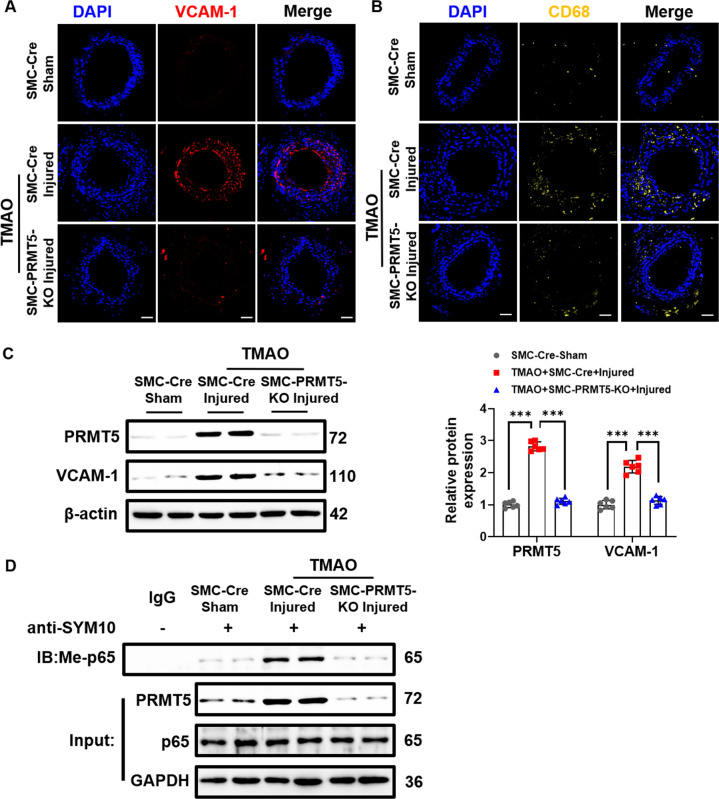
PRMT5 contributes to TMAO induced vascular inflammation in vivo. SMC-PRMT5-KO or SMC-Cre mice were subjected to wire-induced carotid artery injury. TMAO (86 μmol) or saline was administered daily by intraperitoneal injection for 7 days. **A** Representative photomicrographs for VCAM-1 in carotid arteries at 7 days after wire injury. Scale bar, 50 μm. **B** Representative photomicrographs for CD68 in carotid arteries at 7 days after wire injury. Scale bar, 50 μm. **C** Representative blots and quantitative analysis of VCAM-1 and PRMT5. The protein expression level was normalized to β-actin. **D** Representative blots of arginine methylation of p65. Immunoprecipitated SYM10 was blotted with an antibody against p65. Data shown are means ± SD, *n* = 6 per group from three independent experiments. ***P < 0.001 using a one-way ANOVA followed by Tukey’s post hoc test.

Next, we investigated whether PRMT5 contributed to vascular inflammation in vivo using a classical wire injury-induced vascular inflammation model on carotid arteries. SMC-specific PRMT5 knockout (SMC-PRMT5-KO) mice were applied to verify the role of PRMT5 in vascular inflammation (Suppl. Fig. 4). As shown in Suppl. Figure 5, plasma TMAO levels in both SMC-Cre and SMC-PRMT5-KO mice were raised by exogenous TMAO administration. We observed that TMAO treatment significantly increased the expression of VCAM-1 and the macrophage marker CD68 in injured carotids of SMC-Cre mice (Fig. 4A, B). These changes were obviously reduced in TMAO-exposed SMC-PRMT5-KO mice. Western blot analysis further confirmed that expression of VCAM-1 and PRMT5 was greatly increased in TMAO-exposed injured SMC-Cre carotids. In contrast, expression of VCAM-1 was much lower in injured carotids of TMAO-exposed SMC-PRMT5-KO mice than in SMC-Cre mice (Fig. 4C). To further verify whether arginine methylation detected on NF-κB p65 is catalyzed by PRMT5 in vivo, we examined the symmetrical dimethylation of NF-κB p65 in injured arteries of TMAO-exposed SMC-PRMT5-KO mice. SMC-specific PRMT5 knockout resulted in a substantial decrease in arginine methylation levels of TMAO-exposed wire injured carotid arteries (Fig. 4D). Together, these results suggest that PRMT5 regulates VCAM-1 expression via dimethylation of NF-κB p65 on R30.

### Nox4 is involved in TMAO-induced PRMT5 and subsequent VCAM-1 expression

**Fig. 5:**
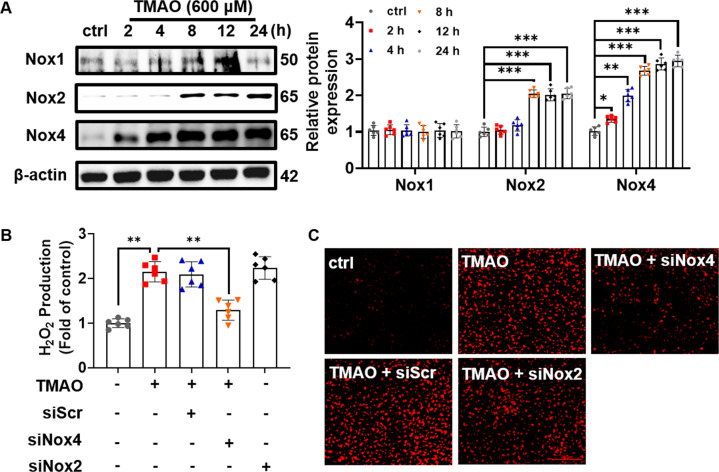
NOX4 knockdown decreases H_2_O_2_ production in VSMC treated with TMAO. **A** Representative blots and quantitative data of Nox1, Nox2 and Nox4 protein expression in TMAO (600 μM) treated VSMC at indicated periods. The protein expression level was normalized to β-actin. **B**, **C** VSMC were treated with scrambled siRNA, Nox1 or Nox4 siRNA, and then treated with TMAO (600 μM). **B** H_2_O_2_ and **C** O_2_^•−^ levels were evaluated by DHE and Amplex Red probe, respectively. Data shown are means ± SD, *n* = 6 per group from three independent experiments. **P < 0.01, ***P < 0.001 using a one-way ANOVA followed by Tukey’s post hoc test.

It has been shown that ROS shape the epigenetic landscape and contribute to VSMC inflammatory responses [15, 16]. Importantly, NADPH oxidase family plays an important role in regulating VCAM-1 expression [17, 18]. To clarify whether NADPH oxidases were involved in TMAO-stimulated PRMT5 and subsequent VCAM-1 expression, the subtypes of NADPH oxidase were analyzed by Western blot. As shown in Fig. 5A, TMAO time-dependently upregulated Nox4 protein expression. In contrast, Nox2 upregulation was delayed, occurring at 8 h post-TMAO stimulation, and its magnitude of upregulation was lower than Nox4 (2.01-fold vs. 2.67-fold for Nox2 upregulation vs. Nox4 upregulation at 8 h). Unlike Nox4 and Nox2, Nox1 expression was not affected by TMAO. In addition, TMAO-induced ROS generation was also measured by DHE staining and Amplex red assay. However, transfection with Nox4 siRNA, but not Nox2 or scrambled siRNA, significantly attenuated TMAO-induced H_2_O_2_ production (Fig. 5B) and O_2_^•−^ levels (Fig. 5C).

**Fig. 6:**
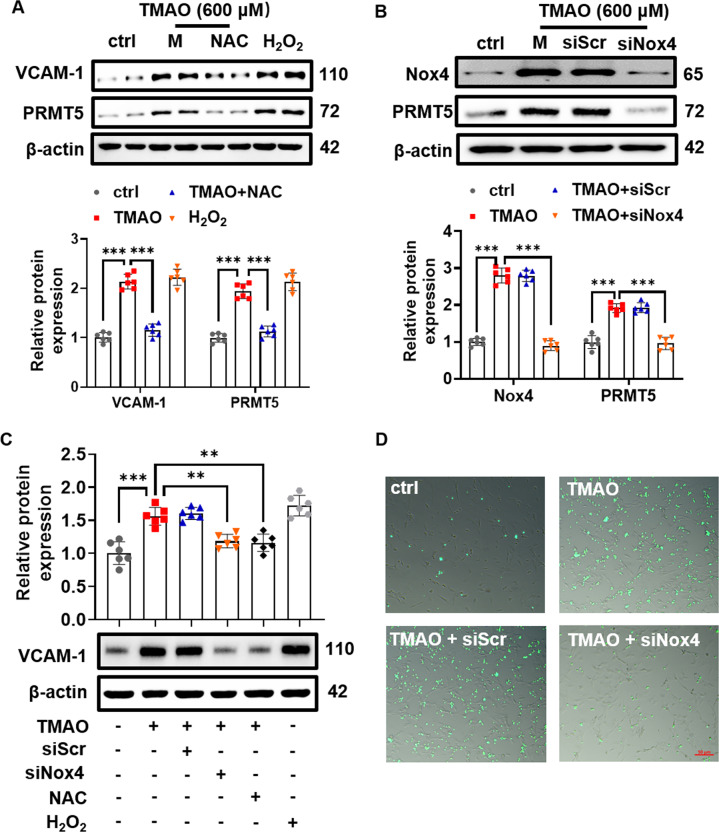
Nox4 knockdown downregulates PRMT5 and VCAM-1 expression. **A** VSMC were pretreated with N-acetyl-cysteine (NAC) and then stimulated with TMAO (600 μM) for 24 h. Representative blots and quantitative analysis of VCAM-1 and PRMT5. The protein expression level was normalized to β-actin. H_2_O_2_ stimulation served as positive control. **B**–**D** VSMC were treated with scrambled siRNA (siScr) or Nox4 siRNA (siNOX4) and then treated with TMAO (600 μM). **B** Representative blots and quantitative analysis of Nox4 and PRMT5. The protein expression level was normalized to β-actin. **C** Representative blots and quantitative data of VCAM-1 protein expression. The protein expression level was normalized to β-actin. H_2_O_2_ stimulation served as positive control. **D** Representative images obtained by fluorescence microscope show cell adhesion. Data shown are means ± SD, *n* = 6 per group from three independent experiments. **P < 0.01, ***P < 0.001 using a one-way ANOVA followed by Tukey’s post hoc test.

We next examined the role of Nox4 in TMAO-induced VSMC inflammation. Pretreatment with N-acetyl-l-cysteine (a nontargeted ROS scavenger, NAC, 1 mM) resulted in a dramatic decrease of TMAO-induced PRMT5 expression, accompanied with reduction of VCAM-1 expression (Fig. 6A). Meanwhile, treatment of VSMC with H_2_O_2_ (50 μM) resulted in enhanced PRMT5 and VCAM-1 expression (Fig. 6A), indicating that H_2_O_2_ was involved in TMAO-induced upregulation of PRMT5 expression. Notably, Nox4 silencing abolished TMAO-induced PRMT5 expression in VSMC (Fig. 6B). Furthermore, TMAO-induced VCAM-1 expression was markedly reduced by pretreatment with NAC (1 mM) for 2 h (Fig. 6C). In contrast, Nox2 knockdown did not affect the expression of VCAM-1 (Suppl. Fig. 6A). Consistent with the results of Nox4 expression, BMDM adhesion was remarkably increased when VSMC were stimulated with TMAO for 24 h (Fig. 6D), while Nox4 silencing, but not Nox2 knockdown, significantly attenuated BMDM adhesion to TMAO-stimulated VSMC (Suppl. Fig. 6B). Collectively, these data indicate that the upregulation of Nox4 contributes to TMAO-induced PRMT5 and VCAM-1 expression in VSMC.

### TMAO-mediated Nox4-NF-YA signaling activation positively regulates PRMT5

**Fig. 7:**
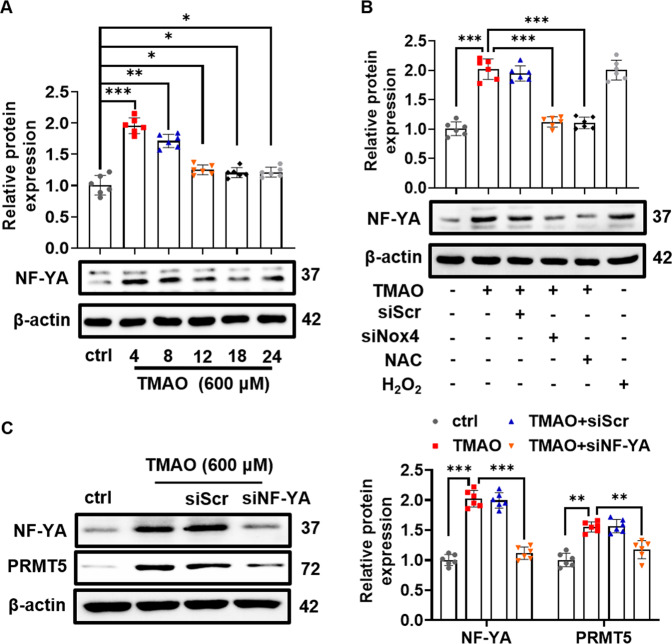
NF-YA is responsible for TMAO-induced PRMT5 expression. **A** VSMC were treated with TMAO (600 μM) for indicated periods. Representative blots and quantitative analysis of NF-YA. The protein expression level was normalized to β-actin. **B** VSMC were transfected with scrambled siRNA (siScr) or Nox4 siRNA (siNox4) and then stimulated with TMAO (600 μM) for 24 h. Representative blots and quantitative analysis of NF-YA. The protein expression level was normalized to β-actin. **C** VSMC were transfected with NF-YA siRNA (siNF-YA) or siScr and then stimulated with TMAO (600 μM) for 24 h. Representative blots and quantitative analysis of NF-YA and PRMT5. The protein expression level was normalized to β-actin. Data shown are means ± SD, *n* = 6 per group from three independent experiments. *P < 0.05, **P < 0.01, ***P < 0.001 using a one-way ANOVA followed by Tukey’s post hoc test.

It is reported that nuclear factor-YA (NF-YA) is critical for PRMT5 expression [19]. As shown in Fig. 7A, incubation with TMAO elicited a peak level of NF-YA expression at 4 h, followed by gradual dropping. Consistent with TMAO-induced expression of PRMT5, Nox4 knockdown, or NAC treatment significantly inhibited TMAO-induced NF-YA expression. H_2_O_2_ treatment alone markedly induced NF-YA expression (Fig. 7B). More importantly, NF-YA knockdown abolished TMAO-induced PRMT5 expression in VSMC (Fig. 7C). Taken together, these results suggested that the Nox4-NF-YA signaling positively regulates PRMT5 expression.

## Discussion

**Fig. 8:**
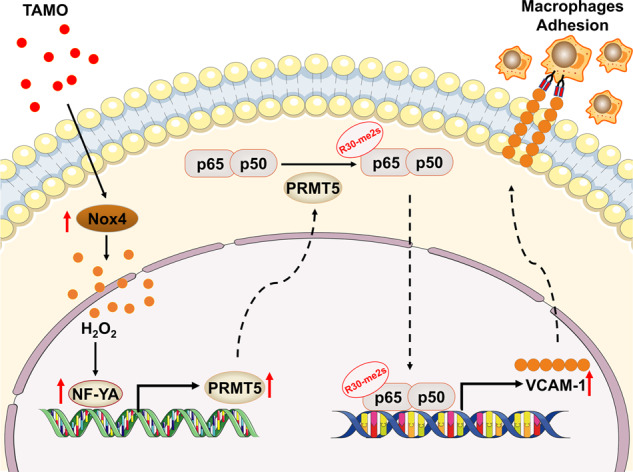
PRMT5 promotes TMAO-induced VCAM-1 expression via methylation of NF-κB p65 in VSMC. H_2_O_2_ generated from the TMAO-stimulated Nox4 upregulation can promote PRMT5 expression and increase methylation of NF-κB p65 on R30, leading to the induction of VCAM-1 and macrophage adhesion.

Several clinical and metabolic studies have demonstrated an association between increased TMAO level and the development of inflammatory vascular diseases [20, 21]. However, the exact mechanisms remain to be explored. In the present study, we show for the first time that the critical role of PRMT5 in TMAO-induced vascular inflammation. PRMT5 knockdown significantly attenuated TMAO‑induced VCAM-1 expression and the adhesion of BMDM to VSMC. Mechanistically, we show that arginine methylation of the NF-κB p65 on R30 homology domain by PRMT5 is critical for TMAO-stimulated induction of VCAM-1 (Fig. 8). Thus, our results highlight the potential of targeting PRMT5 for developing new therapeutic strategies for TMAO-induced vascular diseases.

TMAO, a complex compound derived from phosphatidyl choline/l-carnitine and metabolism by the gut microbiome, has been now recognized as a contributor to inflammatory vascular diseases [22, 23]. In human clinical studies, increased TMAO levels were associated with increased risks of major adverse cardiovascular events [24, 25]. Nevertheless, it has been suggested that TMAO exposure can induce the expression of cytokines and adhesion molecules such as VCAM-1 both in vivo and in vitro [26, 27]. Consistent with these findings, our results showed that TMAO positively regulated VCAM-1 expression in VSMC, leading to increased macrophage adhesion, which plays a vital role in foam cell formation. However, the molecular mechanisms underlying TMAO-induced VCAM-1 expression, and subsequent events remain to be further elucidated. In this regard, the present study for the first time found that TMAO upregulated PRMT5 expression, and PRMT5 upregulation contributed to TMAO-associated VSMC inflammation. By knocking down or overexpressing PRMT5 in VSMC, we showed that PRMT5 was required for VCAM-1 expression and the adhesion of BMDM to TMAO-stimulated VSMC. Our findings agree with an earlier report that aberrant PRMT5 expression is associated with the endothelial cell inflammatory response [13].

There is an emerging body of evidence that PRMT5 plays an important role in modulating transcription by catalyzing symmetric dimethylation on histones [28]. For example, Cao et al. reported that PRMT5 regulated IL-12 expression by arginine modification of histone in innate immunity [29]. PRMT5 can also methylate nonhistone proteins, such as the transcription factors p53 [30], HOXA9 [13], and p65 [31]. Although PRMT5 has been linked with essential cellular processes such as gene regulation, cell proliferation, and differentiation, its role has yet to be described in VSMC inflammatory activation. In this study, we provided unique insights regarding how PRMT5 upregulation might promote VSMC inflammation, i.e., via symmetrically dimethylated NF-κB p65 on arginine 30 (R30). Previous studies have demonstrated that NF-κB p65 is involved in inflammatory activation that occurs at sites of atherosclerotic [32, 33]. During inflammation, NF-κB p65 activation is sufficient for the expression of VCAM-1 [34, 35]. Post-translational modifications of NF-κB p65 are recognized as a primary driver for the context-dependent gene transcription, including phosphorylation, acetylation, lysine methylation, and ubiquitination [36–38]. Accordantly, we found that TMAO promoted NF-κB p65 methylation. Recent studies have shown that plant homeodomain finger protein 20 (PHF20) promotes NF-κB p65 transcriptional activity by interacting with NF-κB p65 in a methylation-dependent manner, which leads to persistent NF-κB p65 phosphorylation [39]. Furthermore, accumulating evidence supports the important role of dimethyl R30 in increasing the transcriptional ability of NF-κB p65 [31, 40]. Similarly, we found that p65 was dimethylated on R30 by PRMT5 following TMAO stimulation, leading to activation of NF-κB p65 responsible for the expression of VCAM-1.

The potential mechanisms of TMAO-induced PRMT5 expression were further investigated in this study. Current advances about epigenetics have revealed a parallel scenario showing the influence of oxidative stress as a major regulator of epigenetic gene regulation via modification of DNA methylation, histones, and microRNAs [41]. Oxidative stress induced by excess ROS production has emerged as a critical contributor to the initiation and progression of vascular diseases [42, 43]. Several recent studies have reported the putative roles of different Nox isoforms in VSMC that generate ROS, and the subsequent repercussions on VSMC proliferation and inflammatory cell migration that contribute to vascular disease progression [44–47]. In this study, we found that Nox4-generated H_2_O_2_ was involved in TMAO-induced upregulation of PRMT5. Besides, Nox4 silencing significantly attenuated BMDM adhesion to TMAO-stimulated VSMC. Consistently, a Nox4/RhoA/focal adhesion kinase-dependent VSMC adhesion has also been recently reported [48].

Taken together, we demonstrate that PRMT5 promotes TMAO-induced inflammatory response in VSMC in vitro and in vivo. This occurs through PRMT5-mediated dimethylation of NF-κB p65 on Arg-30. Overall, our data support targeting PRMT5 as a potential therapeutic strategy for the treatment of TMAO-associated VSMC inflammation and the related diseases.

## Materials and methods

### Animals

All animal procedures conformed to the Guide for the Care and Use of Laboratory Animals published by the US National Institutes of Health (NIH Publication, 8th Edition, 2011) and were approved by the Animal Ethics Committee of Jiangnan University (JN.NO20180615C1440210). SMC-specific PRMT5-KO (SMC-PRMT5-KO) mice were created by crossing PRMT5^flox/flox^ mice (stock No. T008183, GemPharmatech Co. Ltd., Nanjing, China) with SM22α-Cre mice (B6. Cg- Tg [Tagln-cre] 1Her/J; stock No. 017491; Jackson Laboratory). PRMT5^flox/flox^ mice were constructed with the CRISPR/Cas9 system. The primers used for SM22-Cre mice genotyping were as follows: F1: 5′-CTAGGCCACAGAATTGAAAGATCT-3′, R1: 5′-GTAGGTGGAAATTCTAGCATCATCC-3′ and F2: 5′-GCGGTCTGGCAGTAAAAACTATC-3′, R2: 5′-GTGAAACAGCATTGCTGTCACTT. PRMT5^flox/flox^ mice were genotyped using the primers 5′-TTCTCTAGGGCCTTGAAGACTGGG-3′ and 5′- GGGCACATCACTTTCACTACACTGT-3′. Their age-matched and gender-matched littermate SMC-Cre mice on a C57BL/6 background were used as control mice. SMC-PRMT5-KO mice were identified through Western blot analysis.

### Wire injury-induced vascular inflammation model

Male mice (24–28 g, 10–12-week-old) underwent complete carotid artery injury. In brief, the mice were anaesthetized with isoflurane in oxygen. The left carotid artery was carefully exposed, and the external carotid artery was ligated with an 8-0 suture immediately proximal to the bifurcation of the internal and external carotid arteries. Wire-induced denudation injury was achieved by removing the endothelium with a guidewire (0.38 mm in diameter, No. C-SF-15-15; Cook, Bloomington, USA). The guidewire was withdrawn with a rotating motion, and the process was repeated five times. After the guidewire was carefully removed and blood flow was restored. The skin incision was then closed. The sham littermate control mice underwent the same procedures without the vascular injury. SMC-Cre mice were randomly divided into control group (Sham, *n* = 6) and vascular injured model + TMAO group (SMC-Cre + Injured + TMAO, *n* = 6). SMC-PRMT5-KO mice were also used in vascular injured model + TMAO group (SMC-PRMT5-KO + Injured + TMAO, *n* = 6) in this study. TMAO (86 μmol) or saline was administered daily by intraperitoneal injection for 7 days [26]. The TMAO dose used in the present study was based on the dosage reported by the team discovering the relationship between TMAO and cardiovascular disease. All mice were maintained at the Animal Housing Unit of Jiangnan University (Jiangsu, China) under a controlled temperature (23–25 °C) and a 12 h light–12 h dark cycle. At least six independent mice per group from three independent experiments.

Three groups of operated animals were processed for morphological studies at 7 days after the operation, the time corresponding to peak inflammatory cell infiltration in this model [49, 50].

### Cell isolation from rats and cell culture

VSMC were cultured by the explant method as described previously [51]. In brief, thoracic aortas of adult male Sprague–Dawley rats were removed and washed with cold phosphate-buffered saline (PBS). Aortic media were cut into pieces (<1 mm^3^) after removal of the intima and adventitia and then maintained in Dulbecco’s modified Eagle’s medium (DMEM; Hyclone Laboratories Inc., Logan, UT, USA) supplemented with 20% fetal bovine serum (FBS) and 1% penicillin-streptomycin at 37 °C in humidified air with 5% CO_2_. The purity of VSMC was confirmed by positive staining with alpha-smooth muscle actin (α-SMA) antibody. VSMC were used in passages 2–6.

Primary bone marrow-derived macrophages (BMDM) for adhesion assay were obtained from femoral bone marrow suspensions of C57BL/6 mice, and plated at 3 × 10^6^ cells/mL and differentiated for 7 days in the DMEM supplemented with 10% FBS containing macrophage-colony stimulating factor (M-CSF, 20 ng/mL). All cells were regularly tested negative for mycoplasma contamination.

### Cell viability measurements

The 3-(4,5-Dimethyl-2-thiazolyl)-2, 5-diphenyl-2H-tetrazolium (MTT) bromide assay kit (Beyotime, Shanghai, China) was used to measure cell viability as described previously [52]. In brief, VSMC were inoculated in a 96-well microplate and treated with indicated concentrations of TMAO for 24 h or TMAO (600 μM) for the indicated periods. MTT working solution (10 μL/well) was added to the wells, and the plate was incubated at 37 °C for 4 h. Subsequently, formazan crystals were dissolved using 100 μL of formazan dissolution reagent. Viable cells were counted by absorbance measurements with a monochromator microplate reader (BIO-TEK, Winooski, VT, USA) at a wavelength of 570 nm.

### Small interfering RNA and plasmid DNA transfection

To introduce small interfering RNA (siRNA) into VSMC, the cells were plated on 6-well plates at 30–50% confluence before transfection. Transfection of cells with siRNA was performed using Lipofectamine 3000 transfection reagent (Invitrogen, CA, USA), following the manufacturer’s instructions. Two siRNA targeting Nox4 siRNA, PRMT5 siRNA, NF-κB p65 siRNA, and NF-YA siRNA were designed by GenePharma (Shanghai, China). The ones with the highest knockdown efficacy were selected for the following experiments. Sequences of siRNA were described in Suppl. Table S1. Gene silencing was monitored by Western blot assay after 48 h of post-transfection. Protein expression of PRMT5, NF-κB p65, Nox2, Nox4, and NF-YA in TMAO-treated VSMC was effectively attenuated by RNAi-based knockdown (>75%) (Suppl. Fig. 7).

The plasmids pcDNA3.1/Flag-p65^WT^, pcDNA3.1/Flag-p65^R30A^ (replacement of arginine 30 by alanine, R30A) were purchased from GENEWIZ (Suzhou, China). For transfection, adherent VSMC of 60% confluence in 6-well plates were transfected using Lipofectamine 3000 (Invitrogen) according to the manufacturer’s instructions.

### Western blot analysis

Western blot analysis was performed as previously described [53]. Cells were homogenized in ice-cold lysis buffer RIPA containing protease and phosphatase inhibitor cocktail (Sigma-Aldrich, Shanghai, China). Equal amounts (30 μg) of protein were separated by electrophoresis in 10% sodium dodecyl sulfate-polyacrylamide electrophoresis gel and transferred to polyvinylidene difluoride membranes (Millipore, MA, USA). After blocking with 5% nonfat dried milk and then incubated overnight at 4 °C with appropriately diluted primary antibodies. After being washed with Tris-buffered saline (TBS)-Tween 20, the membranes were incubated with horseradish peroxidase-conjugated anti-rabbit (Cat#65-6120) or anti-mouse secondary antibodies (Cat#62-6520) (Thermo Fisher, WA, USA, dilution 1:5000) for 2 h at room temperature, and immunoreactivity was analyzed by Western Lightening Plus enhanced chemiluminescence (Millipore) according to the manufacturer’s instructions. Antibodies for PRMT5 mAb (Cat# ab109451, 1:10,000) and SYM-10 (Cat# 07-412, 1:1000) were purchased from Abcam (Shanghai, China) and Millipore (CA, USA), respectively. Antibodies for Nox1 mAb (Cat# A8527, 1:1000), Nox2 mAb (Cat# A19701, 1:1000), and β-actin mAb (Cat# AC038, 1:50,000) were purchased from ABclonal (Wuhan, China). Antibodies for Nox4 pAb (Cat# 14347-1-AP,), NF-YA pAb (Cat# 12981-1-AP), VCAM-1 mAb (Cat# 11444-1-AP), Flag pAb (Cat# 20543-1-AP), and NF-κB p65 pAb (Cat# 10745-1-AP, all in 1:1000) were purchased from Proteintech (Wuhan, China).

### Immunofluorescence staining

Immunofluorescence staining was performed as described previously [54]. At 7-day post-injury periods, the carotid arteries were harvested after circulation perfusion and fixed with 4% paraformaldehyde dissolved in PBS and paraffin-embedded for immunofluorescence analyses. After antigen retrieval process (Improved citrate antigen retrieval solution, P0083, Beyotime, Shanghai, China), the arterial sections were blocked in PBS with 10% goat serum for 1 h and incubated overnight with VCAM-1 antibody (Cat# A19131, ABclonal, Wuhan, China, 1:100) or CD68 (Cat# A13286, ABclonal, Wuhan, China, 1:100) at 4 °C. The sections were then washed with PBS and incubated with Alexa 555-labeled anti-mouse IgG (Cat#A21236, Invitrogen, USA, 1:200) or Alexa 647-labeled anti-mouse IgG (Cat#4414S, Cell Signaling Technology, USA, 1:200) diluted in PBSB (1% BSA in PBS) for 1 h at room temperature. Cell nuclei were stained with DAPI. Immunofluorescence staining was analyzed in a blinded manner by randomly numbering the samples. The images were captured using a confocal laser scanning microscope (LSM880, Carl Zeiss) and analyzed using ImageJ software (NIH, Bethesda, MD, USA).

### Quantitative real-time PCR (qRT-PCR)

Total RNA was isolated using TRIzol reagent (Life Technologies, MA, USA) and was subjected to reverse transcription using Prime‐Script RT reagent kit (TaKaRa Biotechology, Kyoto, Japan) following the manufacturer’s instructions. SYBR^®^ Green RT‐qPCR was performed using RT‐qPCR system (BIO RAD CFX Connect, CA, USA). The relative mRNA levels were normalized to mRNA levels of β-actin (housekeeping control), and calculations for fold change of each mRNA were made on comparative cycle threshold method (2^−ΔΔCt^). The primers used in this study were provided in Supplementary Table S2.

### Co‐immunoprecipitation

Cells were lysed in RIPA lysate (weak) supplemented with protease inhibitor cocktail and phosphatase inhibitor cocktail after the corresponding treatment and washed with PBS. The cell lysate was centrifuged at 12,000×*g* for 15 min. The supernatant was transferred to a new tube and a portion was reserved for input. Immunoprecipitation was performed by mixing cleared cell lysates with the antibodies or normal rabbit IgG as a negative control overnight at 4 °C on a rotator. Protein A/G plus magnetic beads were then added and incubated at 4 °C for 2 h. After washing three times using lysis buffer supplemented with protease inhibitor, complexes were released by boiling for 5 min in 2× loading buffer and subjected to Western blot analyses.

### Cell adhesion assay

BMDM were labeled by BCECF-AM (10 μM, Beyotime Biotechnology, Shanghai, China) at 37 °C for 1 h in DMEM. The cells were then washed with culture medium and centrifuged. VSMC grown on 12-well plates were transfected with siRNA or plasmid and then stimulated with TMAO (600 μM) for 24 h. After washing twice with PBS, the labeled BMDM (2 × 10^5^ cells/mL) were seeded onto VSMC monolayers and incubated for 1 h in a CO_2_ incubator. The nonadherent BMDM were removed by washing with PBS. The cell adhesion was detected using a fluorescence microscope (Zeiss Axio Vert A1, Carl Zeiss, Niedersachsen, Germany). Six images were taken in each well and the number of bound BMDM was counted per image area.

### Detection of O_2_^•−^ and H_2_O_2_ production in VSMC

TMAO-induced O_2_^•−^ and H_2_O_2_ production were measured by DHE and Amplex Red probes, respectively [55]. Briefly, VSMC were transfected with Nox2 or Nox4 siRNA for indicated times before stimulation of TMAO. After TMAO treatment, cells were washed with PBS and incubated with DHE (10 μM) or Amplex Red (50 μM) for 30 min in the dark at 37 °C. Cells were washed with PBS for three times and the fluorescence density was measured by a Varioskan Flash Multimode Reader (Thermo Scientific, MA, USA) or a Zeiss LSM880 microscope (Zeiss, Gottingen, Germany).

### Quantification of TMAO

Quantification of TMAO in plasma samples was performed using stable isotope dilution liquid chromatography-tandem mass spectrometry (LC-MS/MS, Q Exactive, Thermo Fisher Scientific; MA, USA) and monitored in positive MRM MS mode using characteristic precursor–product ion transitions: *m/z* 76 → 58. The internal standard trimethylamine-d_9_ N-Oxide TMAO (d9-TMAO) was added to plasma samples before protein precipitation and monitored in positive MRM mode at *m/z* 85 → 66 [56]. Various concentrations of TMAO standards and a fixed amount of internal standards were spiked into control plasma to prepare the calibration curves for quantification of plasma analytes.

### Statistical analysis

Sample sizes were determined based on preliminary studies. Animal studies were designed to generate groups of equal size using blinded analyses. No sample was excluded from these analyses. The number of replicates and statistical method were indicated in each figure legend. Quantitative data were expressed as a mean ± standard deviation (SD). All data analyses were performed with GraphPad Prism 8 software (GraphPad, La Jolla, CA, USA). *P* < 0.05 was considered statistically significant. Statistical analyses of differences were performed by the Student’s *t*-test between two groups and one‐way analysis of variance followed by Tukey’s test when comparing among multiple groups. These statistical tests were selected to be appropriate for the normality of distribution and homogeneity of variance.

## Supplementary information

Supplemental Material

Reproducibility Checklist

## Data Availability

The main data supporting the findings of this study are available within the article and its Supplementary Figures. Extra data are available from the corresponding author upon request.
