# Supplementary material for: PRMT5 critically mediates TMAO-induced inflammatory response in vascular smooth muscle cells
Source: Cell Death Dis. 2022 Apr 4;13(4):299. doi: 10.1038/s41419-022-04719-7 (PMC8980010; doi:10.1038/s41419-022-04719-7)
Supplement: Supplementary file 1 — Supplemental Material [file 41419_2022_4719_MOESM1_ESM.docx]

**Supporting information**

**Supplementary Figure 1. TMAO upregulates PRMT5 and VCAM-1 expression in human VSMC.** **(A)** VSMC were treated for indicated concentrations of TMAO for 24 h. Representative blots and quantitative analysis of VCAM-1 and PRMT5. The protein expression level was normalized to β-actin. **(B)** VSMC were treated with TMAO (600 μM) for indicated periods. Representative blots and quantitative analysis of VCAM-1 and PRMT5. The protein expression level was normalized to β-actin. Data shown are means ± SD, n = 6 per group from three independent experiments. ***P* < 0.01, ****P* < 0.001 using a one-way ANOVA followed by Tukey’s *post hoc* test.

**Supplementary Figure 2.** **PRMT5 knockdown reduces TMAO-induced VCAM-1 expression in human VSMC.** VSMC were transfected with PRMT5 siRNA (siPRMT5), then stimulated with TMAO (600 μM) for 24 h. Representative blots and quantitative analysis of VCAM-1 and PRMT5. The protein expression level was normalized to β-actin. Data shown are means ± SD, n = 6 per group from three independent experiments. **P* < 0.05, ***P* < 0.01, ****P* < 0.001 using a one-way ANOVA followed by Tukey’s *post hoc* test.

**Supplementary Figure 3. PRMT5 is responsible for TMAO-induced VCAM-1 expression and inflammatory gene expression.** Quantitative analysis of mRNA level of IL-6 and IL-1β from VSMC analyzed by qPCR. The mRNA expression level was normalized to β-actin. Data shown are means ± SD, n = 6 per group from three independent experiments. ***P* < 0.01 using a one-way ANOVA followed by Tukey’s *post hoc* test.

**Supplementary Figure 4.** **PCR analysis of tail genomic DNA. (A)** PCR for SM22α-Cre recombinase expression. **(B)** PCR for deletion of PRMT5.

**Supplementary Figure 5.** **Plasma levels of TMAO in SMC-Cre and SMC-PRMT5-KO mice by exogenous TMAO administration.** Plasma levels of TMAO were measured at the end of the animal experiment. Data shown are means ± SD, n = 6 per group from three independent experiments. ****P* < 0.001 using a one-way ANOVA followed by Tukey’s *post hoc* test.

**Supplementary Figure 6. The effects of Nox2 knockdown on VCAM-1 expression and TMAO-induced adhesion of BMDM to VSMC.** VSMC were transfected with Nox2 siRNA (siNox2) or scrambled siRNA (siScr) and then stimulated with TMAO (600 μM) for 24 h. **(A)** Representative Western blots and quantitative data of VCAM-1 protein expression. The protein expression level was normalized to β-actin. **(B)** Representative images show that cell adhesion detected by a fluorescence microscope. Data shown are means ± SD, n = 6 per group from three independent experiments. ****P* < 0.001 using a one-way ANOVA followed by Tukey’s *post hoc* test.

**Supplementary Figure 7. The knockdown efficacy of PRMT5, p65, Nox2, Nox4 and NF-YA siRNA. (A-E)** VSMC were treated with scrambled siRNA or PRMT5, p65, Nox2, Nox4 and NF-YA siRNA and then treated with TMAO (600 μM). Representative blots of PRMT5 **(A)**, p65 **(B)**, Nox2 **(C)**, Nox4 **(D)** and NF-YA **(E)**. The protein expression level was normalized to β-actin.

**Suppl. Figure 1**

**
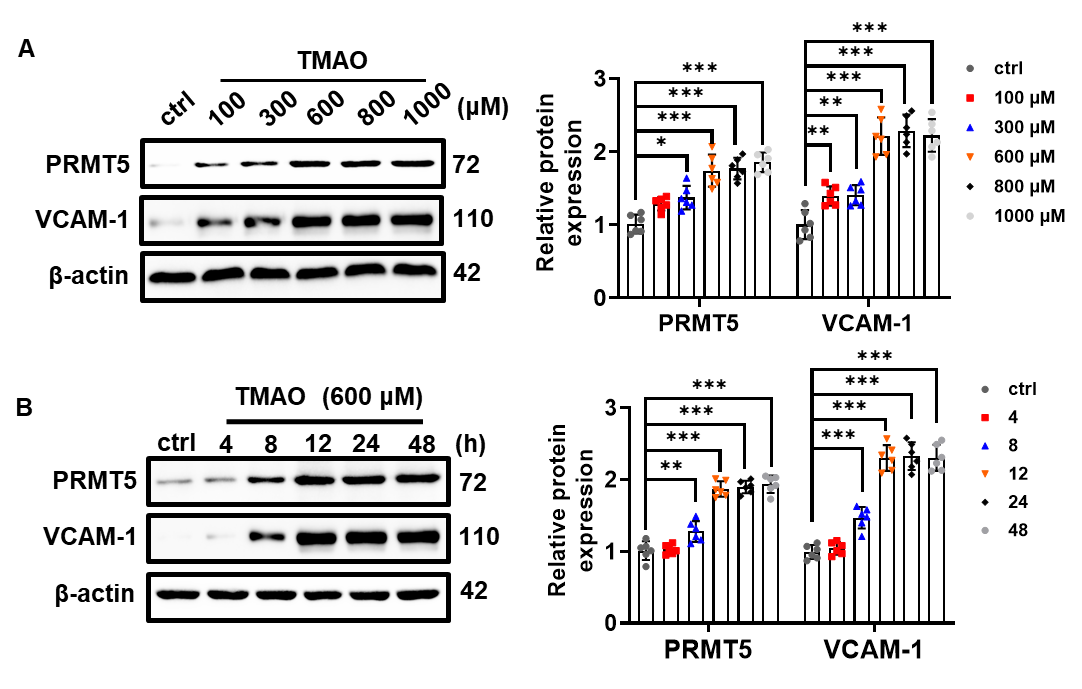
**

**Suppl. Figure 2**

**
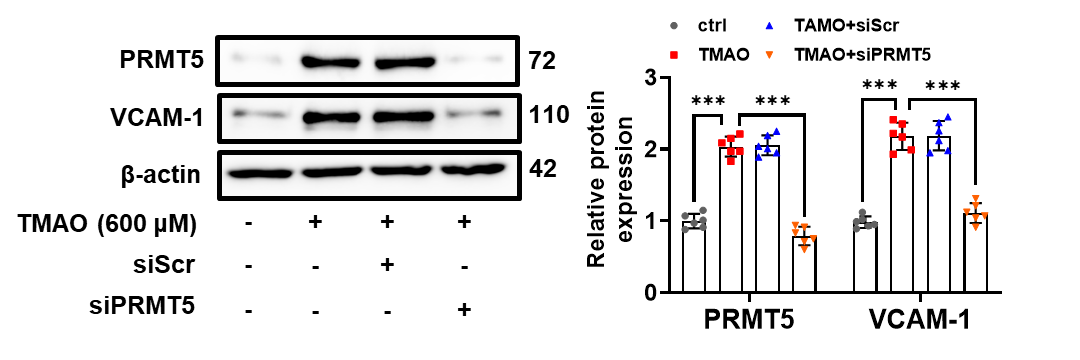
**

**Suppl. Figure 3**

**Suppl. Figure 4**

**
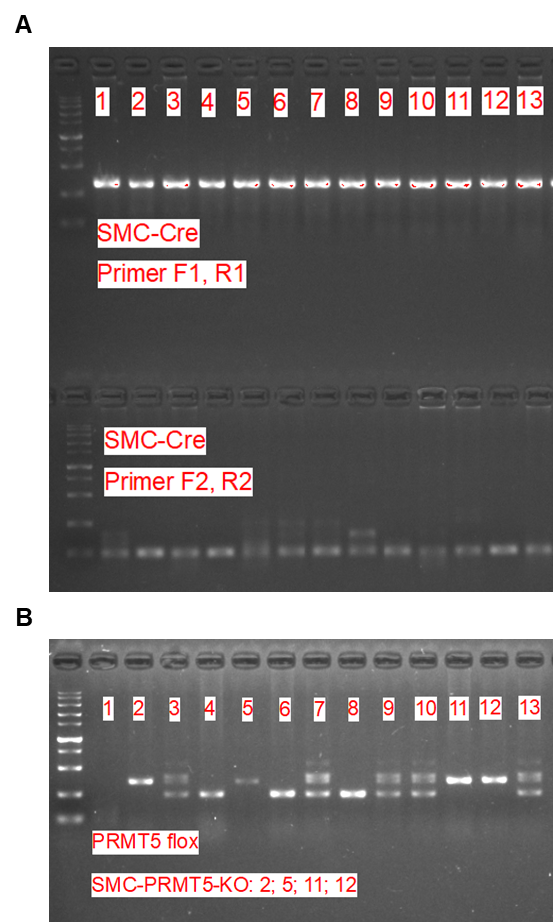
**

**Suppl. Figure 5**

**Suppl. Figure 6**

**
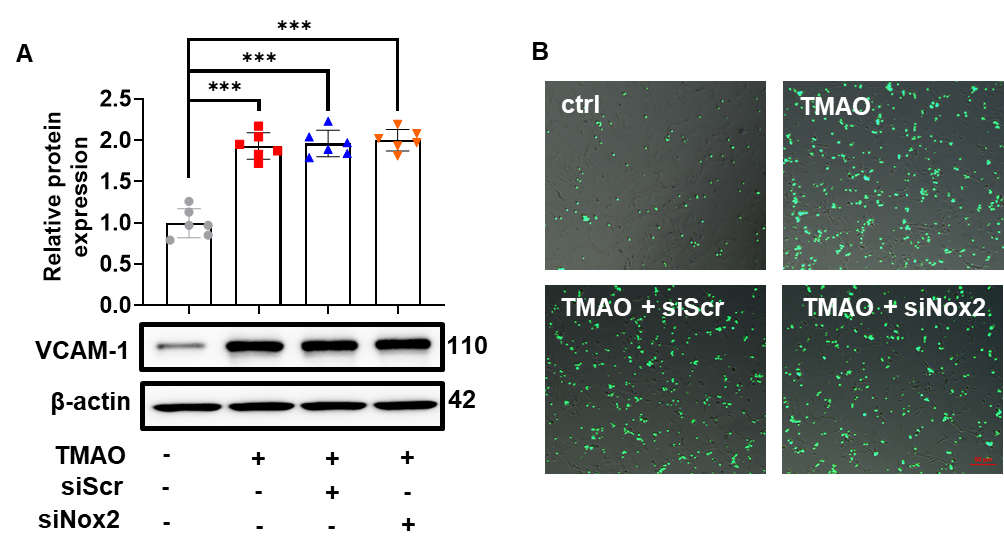
**

**Suppl. Figure** **7**

**
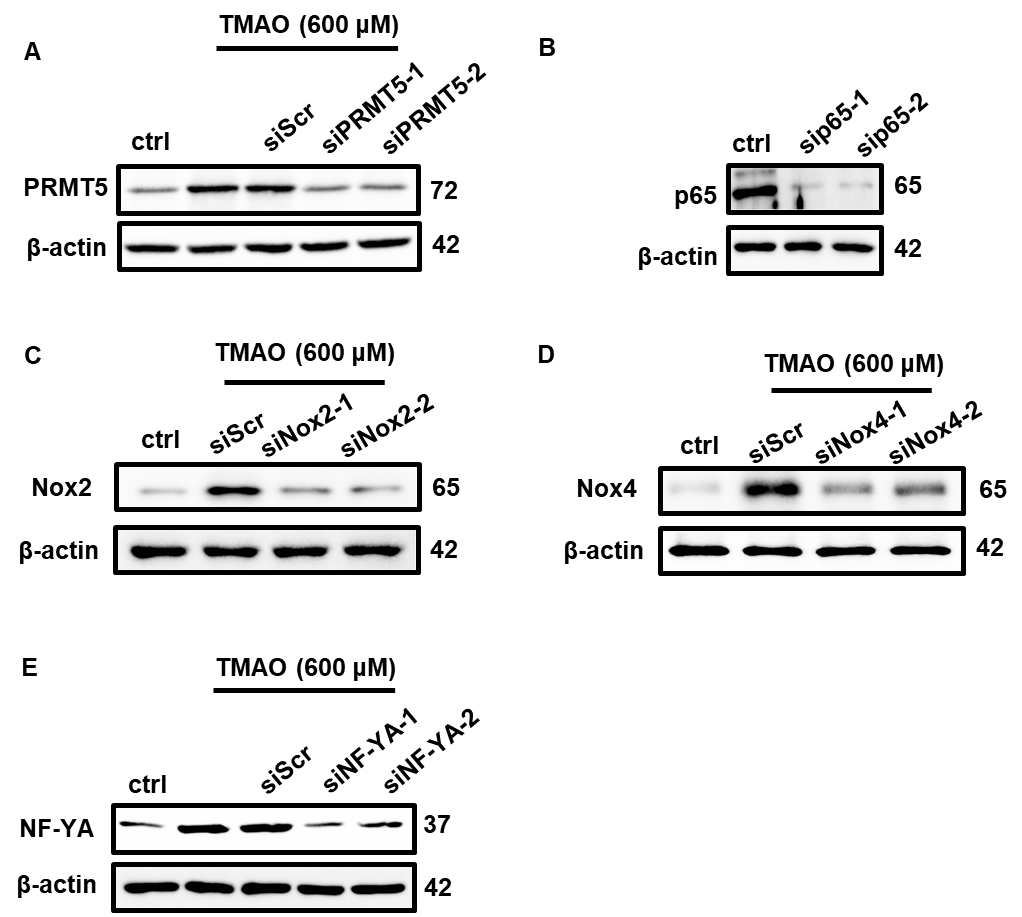
**

**Supplementary Tables**

**Supplementary Table S1.** **siRNA sequences of PRMT5, p65, Nox2, Nox4 and NF-YA used in this study**

| Items | Sequences of siRNA (5’-3’) | |
| --- | --- | --- |
| siPRMT5-1 | sense | CACUCAGAGAAGGAGUUCUTT |
| siPRMT5-2 | sense | GCTAATTGTGGGAAAGCTT |
| sip65-1 | sense | GGCCAUAUGUGGAGAUCAUTT |
| sip65-2 | sense | GGAGUACCCUGAAGCUAUATT |
| siNox2-1 | sense | CCAUGGAGCUGAACGAAUUTT |
| siNox2-2 | sense | CAGUAGCACUCUCUAACAUTT |
| siNox4-1 | sense | GCUUCUACCUAUGCAAUAATT |
| siNox4-2 | sense | GCAACAAACCUGUCACCAUTT |
| siNF-YA-1 | sense | GAAGGGCAGACUAUUGUCUTT |
| siNF-YA-2 | sense | CUCUGUGCCUGCUAUCCAATT |
| Negative control | sense | UUCUCCGAACGUGUCACGUTT |

**Supplementary Table S2.** **Primers used for qRT-PCR**

| Gene | Forward | Reverse |
| --- | --- | --- |
| IL-6 | TCCAGTTGCCTTCTTGGGAC | GTGTAATTAAGCCTCCGACTT |
| IL-1β | TGAGGCTGACAGACCCCAAAAGAT | GCTCCACGGGCAAGACATAGGTAG |
| β-actin | AGAGGGAAATCGTGCGTGAC | CCATACCCAGGAAGGAAGGCT |
